# Relaxation oscillation of borosilicate glasses in supercooled liquid region

**DOI:** 10.1038/s41598-017-16079-w

**Published:** 2017-11-20

**Authors:** W. D. Liu, L. C. Zhang, K. Mylvaganam

**Affiliations:** Laboratory for precision and nano processing technologies, School of Mechanical and Manufacturing Engineering, University of New South Wales, Sydney, NSW 2052 Australia

## Abstract

Most supercooled non-polymeric glass-forming melts exhibit a shear thinning phenomenon, i.e., viscosity decreases with increasing the strain rate. On compressing borosilicate glasses at high temperature, however, we discovered an interesting oscillatory viscous flow and identified it as a typical relaxation oscillation caused by the peculiar structure of borosilicate glass. Specifically, the micro-structure of borosilicate glass can be divided into borate network and silicate network. Under loading, deformation is mainly localized in the borate network via a transformation from the three coordinated planar boron to trigonal boron that could serve as a precursor for the subsequent formation of a BO_4_ tetrahedron, while the surrounding silicate network is acting as a stabilization/relaxation agent. The formation of stress oscillation was further described and explained by a new physics-based constitutive model.

## Introduction

The nonlinear rheology of glassy materials is at the very centre of non-crystalline physics and mechanics^1–4^, which represents the complex deformation mechanisms of a variety of advanced amorphous materials including soft-matter colloidal suspensions, polymer melts, gels, forms, structural glasses as well as metallic glasses^4,5^. Although significant emphasis has been dedicated in recent years to the dynamic heterogeneity^5,6^, Phillips-Thorpe Rigidity Theory^7–11^, spatial collective flow^2,3,5^ and jamming^1,12,13^ in simple glassy systems, studying the nonlinear rheology of complicated structural glasses remains challenging.

The rheological nonlinearities of structural glasses arise from the metastability, topological rigidity and heterogeneity of the network structure^7–11^ and its relaxation^14–17^. According to the classic Maxwell equation *η* = *Gτ* ^18^, the viscosity *η* of a glass in its supercooled liquid region (SLR) is directly related to the infinite frequency shear elastic modulus *G* and the shear relaxation time *τ*. If the temperature is high, the small relaxation time can quickly release the dynamic heterogeneity of structure produced by external loading, which is characterized as a Newtonian fluid. However, if the temperature approaches the material’s glass transition temperature (*T*_g_), the unrelaxed dynamic heterogeneity will lead to rheological nonlinearity, i.e., non-Newtonian. Studying the nonlinear rheology of structural glass in its SLR is critical, not only for gaining the fundamental understanding of glass science, but also for improving the many advanced techniques used in the production of ultra-precision glass components^19^.

According to the dependence of strain rate, the non-Newtonian liquid can be further divided into two groups: shear *thickening* flow if the viscosity increases with strain rate, and shear *thinning* flow if the viscosity decreases with strain rate^20^. Generally, traditional silicate melts have a shear thinning flow^21^, while shear thickening flow was only found in some colloid glasses or suspensions^20^. Lubchenko stated that^22^ all deeply super-cooled non-polymeric fluids, independent of their chemical details, should exhibit simple shear thinning. This statement was further supported by recent experimental results covering a wide range of inorganic glass-forming liquids including Na_2_Si_2_O_5_, Na_2_Si_3_O_7_, Na_2_Si_4_O_9_, K_2_Si_3_O_7_, K_2_Si_4_O_9_, Li_2_Si_4_O_9_, CaMgSi_2_O_6_ etc^21^. However, all the above glass-forming liquids only have one glass network-former (Silicon).

Borosilicate glass is a type of glasses with silica and boron oxide as the main glass-forming networks. The material is well-known for its very low thermal expansion coefficient, weak electrical conductivity, high resistance to thermal shocks and good stability in terms of corrosion^23–26^. The newly developed low-*T*_g_ Borosilicate glass is a material suitable for making precision lenses by thermoforming. Due to the anomalous influence of borate in the network, the thermo-mechanics and dynamics of borosilicate glasses are very different with those of traditional silicate glasses, as revealed by many experimental and numerical results^27–29^. For example, the borosilicate network is constituted from 4-fold coordinated Si and 3- and 4-fold coordinated boron^27,30–33^. With increasing the temperature, rigid 4-fold coordinated boron transforms to softer 3-fold coordinated boron. This transformation also contributes to the viscous flow of borosilicate glass^31^. It is thus expected that this type of materials can have more complicated rheological features. However, most studies were only on the changes of viscosity influenced by compositions and thermal processing^27–29^.

This paper aims to investigate the nonlinear rheology of borosilicate glass in its supercooled liquid region. To this end, high-temperature uniaxial compression tests will be performed over a wide range of loading conditions. Moreover, the underlying microstructural features and viscous flow mechanisms of borosilicate glass will be discussed. Finally, a physics-based constitutive model will be established for describing the nonlinear rheology of the material.

## Results and Discussion

### High-temperature uniaxial compression

**Figure 1:**
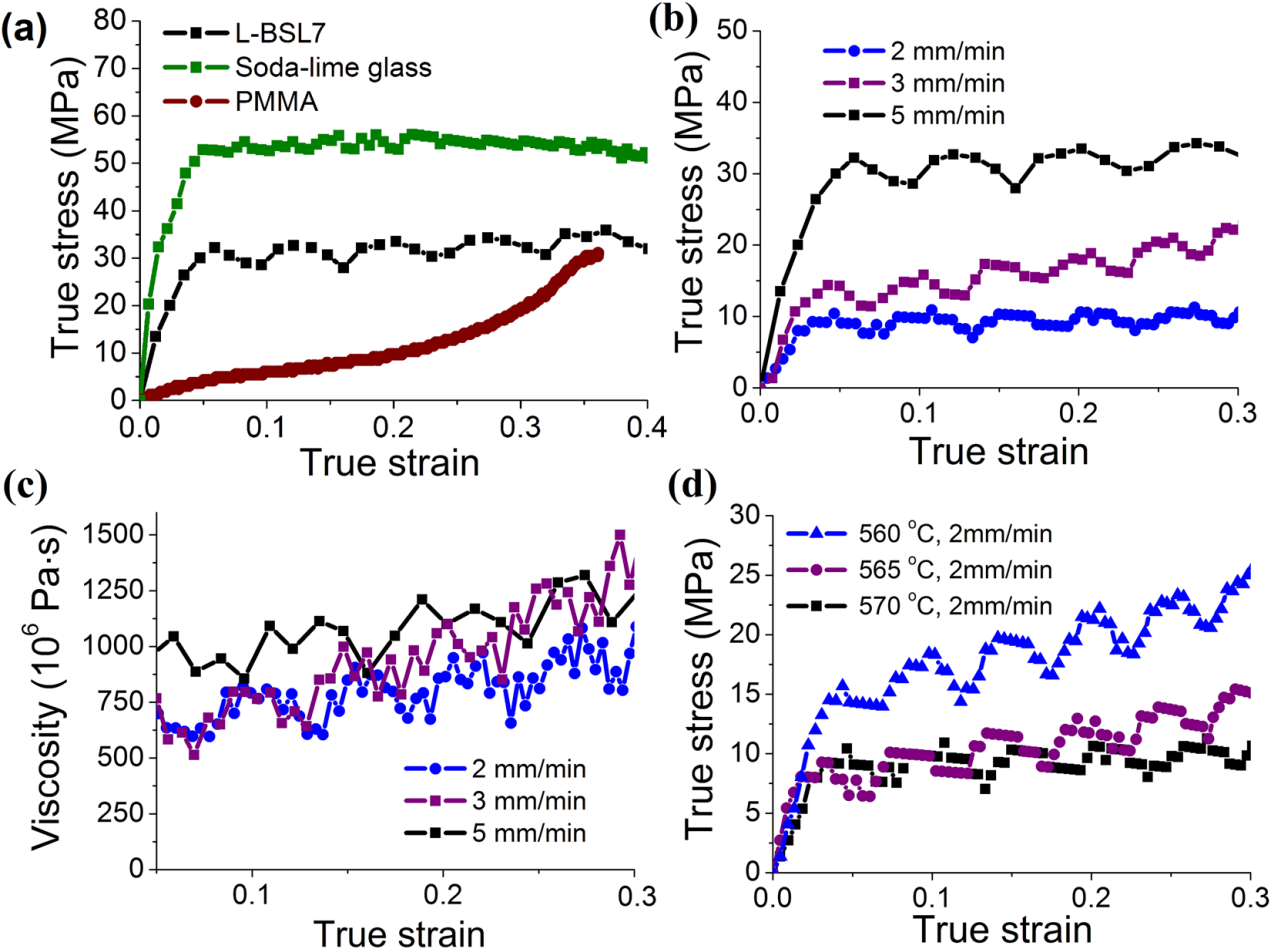
(**a**) Stress-strain curves of L-BSL 7, soda-lime glass and PMMA at loading speed 5 mm/min. (**b**) The stress-strain curves of L-BSL at three different compressive speeds and (**c**) the corresponding viscosity-strain curves. (**d**) The stress-strain curves of L-BSL 7 at three different temperatures.

In this study, a borosilicate glass L-BSL 7 (*T*_g_ ~ 498 °C) was selected and a series of high-temperature (above *T*_g_) uniaxial compressing tests were conducted in precision glass moulding machine. The details can be found in the section of Methods. Figure 1a presents the true stress-strain curve of L-BSL 7 at a loading speed of 5 mm/min and a temperature of 570 °C. Initially, the stress increases from zero to 32 MPa linearly. After yielding, the stress presents an oscillation with an amplitude of 5 MPa and a characteristic strain increment of 0.06 in one oscillation cycle. To exclude artificial factors such as machine controlling etc., the rheology of soda-lime glass (SiO_2_-Na_2_O-CaO) and PMMA (Polymethylmethacrylate) were tested using the same machine. Considering that the *T*_g_ of soda-lime glass and PMMA are different from that of borosilicate glass, the test temperatures were set at 630 °C and 105 °C for them, respectively, to achieve a similar loading force with that of L-BSL 7. Their stress-strain curves shown in Fig. 1 clearly indicate that there is no fluctuation, proving the oscillation is not induced by machine control.

The strain rate effect on the oscillation was studied under three different loading speeds (2 mm/min, 3 mm/min and 5 mm/min), and the stress-strain curves are shown in Fig. 1b. Clearly, the amplitude of the oscillation increases with increasing compressive speed. However, it is interesting to note that the characteristic strain of oscillation is almost the same. The corresponding nominal viscosities after yielding (Fig. 1c) can be estimated from $\eta =\sigma /3\dot{\varepsilon }$, where $\sigma$ and $\dot{\varepsilon }$ is uniaxial stress and strain rate, respectively. As shown in Fig. 1c. Both the amplitude and period of the oscillation of viscosities are almost the same. The higher the compressive rate, the larger the viscosity is. This is a feature of shear thickening phenomenon, which is unexpected for non-polymeric super-cooled fluids^22^.

To further explore the observed oscillation phenomenon, similar uniaxial compressive tests were carried out at two other temperatures (565 °C and 560 °C), with the same loading speed of 2 mm/min. Similar stress oscillations were obtained as shown in Fig. 1d. The amplitude of oscillation increases with decreasing temperature, but the characteristic strain doesn’t change. The lower the temperature, the higher the viscosity, and thus the higher the flow stress is.

**Figure 2:**
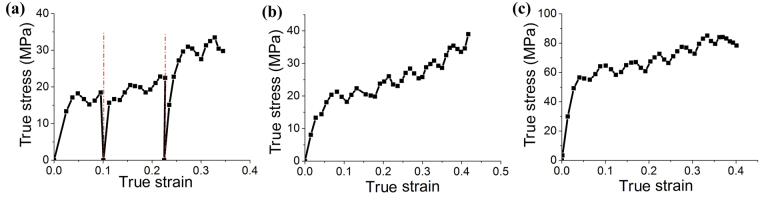
(**a**) Stress-strain curve during a typical loading/unloading test of L-BSL 7 at a temperature of 570 °C. Stress-strain curves of (**b**) an annealed L-BSL 7 and (**c**) a new borosilicate glass P-BK7.

To study whether the stress oscillation is influenced by loading history, cyclic load/unload tests were carried out at 570 °C. Briefly, the specimen was heated from room temperature to target temperature (570 °C); after 10 minutes thermal soaking at 570 °C, the specimen was compressed to 1 mm shorter with a speed of 1 mm/min. Then the specimen was unloaded and cooled down to room temperature. After that, another cycle was carried out. It is found that after unloading the reloading follows the previous trend of oscillation as shown in Fig. 2a. It means that the oscillation is not a simple dynamic event, but can be traced to underlying structure issues.

To check whether the fluctuation is induced by the initial structural heterogeneity and/or phase separation^34^, an annealed sample of L-BSL 7 was prepared by keeping the sample in a furnace at a temperature of 570 °C for 10 hours. The annealed specimen was also polished and etched by very weak acid solutions (0.5% HF in water)^34^ for 24 hours. Presence of any significant phase separation can be identified from the disappearance of the soft borate phase that would be etched away by the acid. However, no such evidence was found in the scanning electron microscope. Figure 2b presents the stress-strain curve of the annealed sample at 570 °C. This curve is very similar to those before annealing, which also excludes the possible influence of initial structural heterogeneity, water impurities and/or phase separation. Moreover, a new borosilicate glass P-BK7 (with a similar composition with L-BSL 7) provided by another supplier Schott, also demonstrated the same phenomenon, as shown in Fig. 2c. The characteristic period of strain is about 0.06, the same with that of L-BSL 7 glass. All of these prove that this stress oscillation should be an intrinsic property of borosilicate glasses.

Above results indicate that the stress oscillation under uniaxial compressive loading is an inherent mechanical behaviour of borosilicate glass melts. To our knowledge, this oscillation has never been found in common silicate glasses. In the SLR of silicate glasses, shear thinning is proposed to be a common rheological feature^22^. It is because with increasing strain rate the shear viscosity is dramatically reduced owing to an increase in the structural relaxation rate^22^. Specifically, the high viscosity in super-cooled melts arises from the transient structures that last longer than hundreds of atomic vibration periods. Thus, the viscosity of a super-cooled fluid is proportional to the average structural relaxation time. To accommodate external force, the immediate environment of the compressive region will relax faster than the surrounding material. A shorter structural relaxation time implies a lower viscosity, and hence leads to shear thinning. Apparently, borosilicate glass studied in this paper does not follow this scenario.

### Relaxation oscillation

Interestingly, similar stress oscillation phenomena have been observed in other materials, which is due to the coexistence of localized deformation and surrounding structural relaxation^35–41^. For example, shear thickening suspensions can produce S-shaped stress-strain rate curve with an unstable branch^35^. The existence of this unstable branch leads to cycles of alternation between the thickened state and the relaxed state, i.e. shear thickening oscillation. Oscillated or serrated plastic flow has been observed in many solution-hardening-alloys such as Ni-C alloy, known as Portevin–Le Chatelier effect^36–40^. It has been well accepted that the serrated plastic flow is caused by dynamic strain hardening. Quasi-periodic avalanche bursts have been observed in slowly compressed nickel microcrystals^41^. The periodic plastic flow arose from the competition of fast avalanche glides and slow relaxation processes. Moreover, it was found that the period in strain scale didn’t change with strain rate, which is very similar to our observation^41^.

To reveal the underlying mechanism of stress oscillation observed in borosilicate glasses, it is essential to understand the microstructure of borosilicate glass and the possible dynamic processes. Nuclear Magnetic Resonance (NMR) showed that the structure of borosilicate glass consists of borate-rich and silicate-rich networks, with randomly distributed alkali cations, although the network generation details were not given^27,30–33^. However, our ab initio analysis using the density functional theory as shown in Supplementary Information does show such mechanisms. The borate network composed of four-coordinated BO_4_ units and three-coordinated BO_3_ units^31^. On thermal/mechanical loading the changes in structure and properties of borosilicate glass are mainly achieved through the exchange of the two boron speciation via the reaction below

1 $${{\rm{BO}}}_{4}\mathop{\longleftrightarrow }\limits^{{{\rm{M}}}^{+}}{{\rm{BO}}}_{{\rm{3}}}+{\rm{NBO}}$$

where NBO is non-bridging oxygens, M^+^ is Alkali ion to balance the partial negative charge on the bridging oxygens^31^. The viscous flow of borosilicate glass can therefore be achieved via the exchange of the basic units, both in borate domain and silicate domain^31^. In borosilicate glass, an NBO under the loading condition can react with a BO_3_ group to form a BO_4_ unit, which could serve as a temporary intermediate state in viscous flow. The newly formed BO_4_ can break up to form a new NBO^27,31^.

The first-principle studies by Pedesseau *et al*.^28^ showed that in borosilicate glass the transformation of BO_3_ units into BO_4_ is more complicated than the above-mentioned reaction where the transformation must involve a simultaneous change of the silicate sub-network. Edwards *et al*.^42^ reported recently that under an isotropic stress, an anisotropic elastic deformation of the BO_3_ planar triangle transforms to a trigonal pyramid that could serve as a precursor for the subsequent formation of a BO_4_ tetrahedron. We further performed all electron density functional theory calculations using Gaussian 09 code to locate the transition state and thereby to work out the activation energy for the conversion of BO_3_ to BO_4_ through reaction with non-bridging oxygen of the SiO_4_ unit. The first-principles density functional theory calculations using B3LYP functional and 6-31 G** basis set showed that the transition state had one negative frequency that corresponds to the transformation of BO_3_ (planar) to BO_3_ (trigonal) and the entering of non-bridging oxygen of the SiO_4_ unit with the B–NBO distance of 2.02 Å, leading to the formation of BO_4_ tetrahedron. The overall reaction is endothermic and the activation energy is only 54.8 kcal/mol confirming the transformation of BO_3_ to BO_4_ involving a simultaneous change of silicate net-work is favourable.

This newly formed BO_4_ group can break up to form a new non-bridging oxygen bonded to another silicon unit as proposed by Stebbins and Ellsworth^31^. Considering the stability of silicate network during deformation, the fast deformation-induced phase transformation in borate network can be relaxed slowly by the surrounding silicate network. Thus the observed oscillatory viscous flow could be the competitive outcome of the deformation and relaxation in these two networks.

### Constitutive modelling of borosilicate glass

It is noted that above relaxation oscillation phenomena can be characterized by similar mathematic equations^35,36,41^, although their underlying physical mechanisms are different. This type of equations is known as van der Pol oscillator or FitzHugh-Nagumo models in nonlinear physics^43^. Based on the concept of these models, we developed a constitutive model for borosilicate glass to describe the observed stress oscillation. This model consists of three parts as summarized in equations (2–4): (1) the relationship between stress rate $\dot{\sigma }$ and strain rate $\dot{\varepsilon }$, (2) the changes of $\dot{\varepsilon }$ with the waiting time of viscous flow unit *t*_a_ and (3) the evolution of *t*_a_. The details of this model can be found in Methods section.

2 $$\dot{\sigma }=K(R-\dot{\varepsilon })$$

3 $$\ddot{\varepsilon }=\dot{\varepsilon }\{\frac{KR}{{\rm{\Phi }}}-\frac{K}{{\rm{\Phi }}}\dot{\varepsilon }-\frac{\psi \alpha }{{t}_{c}}\exp \{-{(\frac{{t}_{a}}{{t}_{c}})}^{\alpha }\}{(\frac{{t}_{a}}{{t}_{c}})}^{\alpha -1}{\dot{t}}_{a}\}$$

4 $${\dot{t}}_{a}=1-\frac{\dot{\varepsilon }}{{\rm{\Omega }}}{t}_{a}$$

where *K* is the nominal modulus, *A* and *l* are the cross-sectional area and length of the specimen, *R* is ratio of the compressive speed *V* to *l*, *Φ* and *Ψ* are two parameters relating the activation energy dependence on the excess stress and on the state variable, *t*_*c*_ is the characteristic diffusion time depending on the interaction between borate network and silicate network, *α* is a correction factor, *t*_a_ during the waiting time of viscous flow unit and its average value at a constant strain rate is ${\bar{t}}_{a}$, and *Ω* is the average characteristic strain produced by viscous flow units.

**Figure 3:**
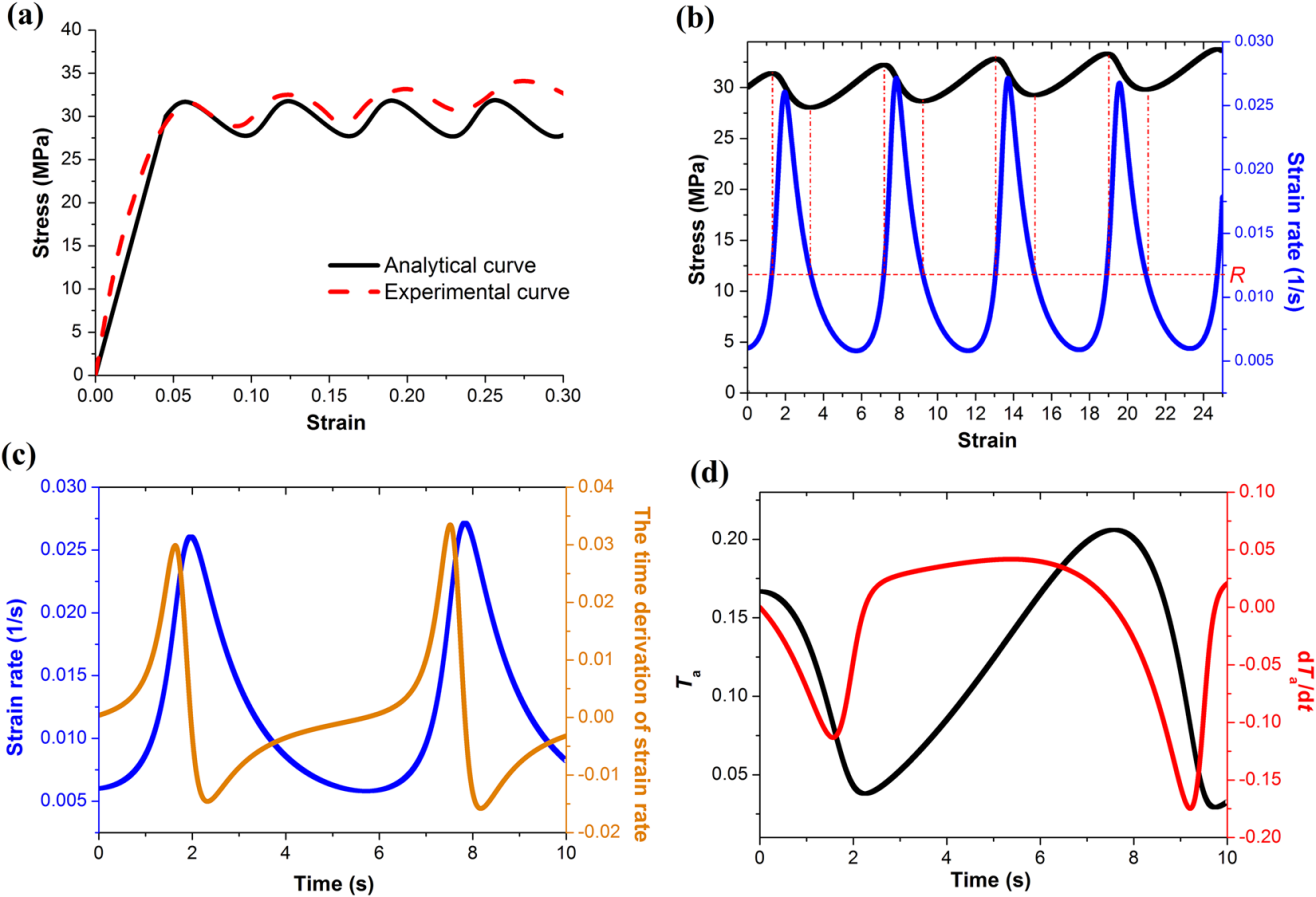
(**a**) Stress oscillation predicted by the model. (**b**) The comparison of the evolutions of stress and strain rate. (**c**) The comparison of the evolutions of strain rate and its time derivation. (**d**) The comparison of the evolutions of *T*_a_ and its time derivation d*T*_a_/d*t*.

Figure 3a presents the stress oscillation (*T* = 570 °C, loading speed 5 mm/min) predicted by the developed constitutive model. Overall good agreement between the analytical curve and the experimental result was obtained. This model doesn’t consider the interface friction effect, and thus the increasing trend of the experimental curve was not captured. According to equation (2) and Fig. 3b, the observed stress oscillation is attributed to the periodic changes of strain rate. When strain rate is larger than *R*, the derivation of stress becomes negative and stress started to decrease. When the strain rate is smaller than *R*, the stress starts to increase. The periodic variation of strain rate arises from the competition of $\dot{\varepsilon }$ and *t*_*a*_ controlled by equations. (3) and (4). Specifically, at the beginning of yielding, the viscous strain rate increases from a very low value to catch up with the high external loading speed. It triggers a self-enhance process of $\dot{\varepsilon }$ via two ways (see Fig. 3c). First, according to equation. (3) the increasing $\dot{\varepsilon }$ enhances its time derivative of $\ddot{\varepsilon }$, which in turn enhance $\dot{\varepsilon }$ itself. Second, the increase of strain rate leads to a negative value of the rate of *t*_a_ and thus the decrease of *t*_a_. The small value of *t*_a_ can produce a very large value of $\ddot{\varepsilon }$ and thus, in turn, enhance the strain rate again. This corresponds to a typical shear thinning process occurring in the borate network. When *t*_a_ becomes very small, however, the decreasing trend of ${\dot{t}}_{a}$ becomes slow and finally changed from negative to positive. This sign change brings about two consequences. First, the third term of Eq. (3) changed its sign immediately and lead to a sudden decrease of $\ddot{\varepsilon }$, and thus $\dot{\varepsilon }$ starts to decrease (see Fig. 3c). That means the trend of shear thinning was stopped by the slow relaxation time. Second, *t*_a_ starts to increase faster and faster because of the continous increase of ${\dot{t}}_{a}$(see Fig. 3d). Finally ${\dot{t}}_{a}$ approaches a stable positive value. A further increase of *t*_a_ makes ${\dot{t}}_{a}$ decrease again and another cycle started. That is the formation mechanism of the observed stress oscillation.

## Conclusion

An interesting, unexpected stress oscillation phenomenon was observed on compressing borosilicate glasses in its supercooled liquid region. This stress oscillation demonstrated a constant characteristic strain, which was independent of strain rate, temperature, and loading and annealing histories. The observed oscillation was further revealed to be a typical relaxation oscillation phenomenon, arising from the competition of the deformation-induced phase transformation in the borate-rich network and the structure relaxation in the silicate-rich network. A constitutive model was developed for describing and understanding this oscillatory viscous flow.

## Methods

### High-temperature uniaxial compression test

A borosilicate glass L-BSL 7 (Ohara GmbH) with *T*_g_ 498 °C and composition of SiO_2_(69.13)-B_2_O_3_(10.75)-Na_2_O(10.40)-K_2_O(6.29) was selected. The cylindrical samples of L-BSL 7 prepared for uniaxial compression test were of the diameter of 3 mm and length of 6.5 mm. A series of uniaxial compressive tests with a constant compressive speed (2 mm/min, 3 mm/min and 5 mm/min) were conducted at constant temperatures of 570 °C in a precision glass moulding (PGM) machine, Toshiba GMP-211. In brief, a glass sample was placed in the furnace of PGM machine and then heated up to 570 °C. A further soaking step was conducted for 10 miniatures to achieve a uniform temperature distribution throughout the sample. After that, the sample was compressed uniaxially by the crosshead in the furnace with a constant speed. The uniaxial stress-strain curve can thus be obtained from the recorded force and displacement.

To study the temperature effect, another two tests were conducted at 560 °C and 565 °C, respectively, with the compressive speed of 2 mm/min. To eliminate the effect of possible motion error of the glass moulding machine, uniaxial compressive tests with a loading speed of 5 mm/min were also carried out for a soda lime glass (SiO_2_-Na_2_O-CaO, *T*_g_ = 564 °C) and an optical polymer PMMA (Polymethylmethacrylate, *T*_g_ = 105 °C) in the same loading range. To exclude the residual stress effect of sample preparation, one L-BSL 7 sample was annealed for 10 h at 570 °C and then compressed with the loading speed of 5 mm/min. To study the loading history effect, loading-unloading cycles were carried out at 570 °C with the loading speed of 5 mm/min. To further verify the oscillation phenomena, a similar borosilicate glass of P-BK7 (SiO_2_(69.13)-B_2_O_3_(10.75)-Na_2_O(10.40)-K_2_O(6.29)) from a different supplier (Schott) was also tested at 570 °C with the loading speed of 1 mm/min. Before loading, all the samples had been heated to the target temperature and preserved for more than 10 minutes to ensure that the material had the same temperature throughout. Details of the loading conditions are listed in Table 1.

**Table 1:** Parameters of uniaxial loading tests.

Test No.	Sample	Temperature	Loading speed
1	L-BSL 7	570 °C	5 mm/min
2	L-BSL 7	570 °C	3 mm/min
3	L-BSL 7	570 °C	2 mm/min
4	L-BSL 7	565 °C	2 mm/min
5	L-BSL 7	560 °C	2 mm/min
6	L-BSL 7 (Step loading-unloading)	570 °C	1 mm/min
7	PMMA	105 °C	5 mm/min
8	Soda-lime glass	630 °C	5 mm/min
9	Annealed L-BSL 7	570 °C	5 mm/min
10	P-BK 7	570 °C	5 mm/min

### Constitutive modelling

The visco-plastic flow in supercooled borosilicate glass is basically the outcome of a biased accumulation of local strain in borate network and a structural relaxation via the interaction of borate network and silicate network. The local strain created by the transformation from the three coordinated planar boron to trigonal boron can be taken as a thermally activated process^44^,

5 $$\dot{\varepsilon }={\dot{\varepsilon }}_{0}\cdot \exp (-{\rm{\Delta }}G/kT)$$

where ${\rm{\Delta }}G$ is the free enthalpy of activation, *T* is temperature, ${\dot{\varepsilon }}_{0}$ is a reference strain rate, and *k* is the Boltzmann constant. ${\rm{\Delta }}G$ is affected by the external stress and the internal local microstructure state near the viscous flow unit. To describe the local microstructure state and its relaxation, the well-accepted concept of free volume *v*_*f*_ was introduced in this model. ${\rm{\Delta }}G$ can thus be expressed as

6 $${\rm{\Delta }}G={\rm{\Delta }}G(\sigma -{\sigma }_{f},{v}_{f})$$

where *σ* is the applied stress and *σ*_*f*_ is the average flow stress at 0 K for a given *v*_*f*_. It is noted that free volume can be created during the activation of viscous flow event and annihilated via the interaction of borate network and silicate network during the waiting time of viscous flow event *t*_a_. For convenience, a non-dimensional state variable ${\bar{v}}_{f}$ was introduced instead of *v*_*f*_ in the model, varying from 0 to 1, i.e.

7 $${\bar{v}}_{f}=1-\exp (-{({t}_{a}/{t}_{c})}^{\alpha })$$

where *t*_*c*_ is the characteristic relaxation time depending on the interaction between borate network and silicate network, *α* is a correction factor. If the strain rate is constant, *t*_a_ approaches its steady state value, ${\bar{t}}_{a}$, where

8 $${\bar{t}}_{a}={\rm{\Omega }}/\dot{\varepsilon }$$

Here, *Ω* is the average characteristic strain produced by viscous flow events. When there is a change in strain rate, *t*_a_ relaxes to its new steady state, and the process can be expressed by a simple first-order relaxation^39^

9 $${\dot{t}}_{a}=({\bar{t}}_{a}-{t}_{a})/{\bar{t}}_{a}$$

We further introduced two additional parameters by linearizing the activation energy dependence on the excess stress and on the state variable ${\bar{v}}_{f}$

10 $$\frac{1}{{\rm{\Phi }}}=-\frac{\partial }{\partial (\sigma -{\sigma }_{f})}(\frac{{\rm{\Delta }}G}{kT})={\frac{\partial \mathrm{ln}\dot{\varepsilon }}{\partial \sigma }|}_{{\bar{v}}_{f}=const}{\rm{and}}\,\psi =\frac{\partial }{\partial {\bar{v}}_{f}}(\frac{{\rm{\Delta }}G}{kT})=-{\frac{\partial \mathrm{ln}\dot{\varepsilon }}{\partial {\bar{v}}_{f}}|}_{\sigma =const}$$

Together with equations (7), (8), (9), one can get the stress-strain rate relation in the following form

11 $$\dot{\varepsilon }={\dot{\varepsilon }}_{0}\,\exp \{\frac{\sigma -{\sigma }_{f}}{{\rm{\Phi }}}-\psi [1-\exp (-{(\frac{{t}_{a}}{{t}_{c}})}^{\alpha })\}$$

and the evolution equation of *t*_a_

12 $${\dot{t}}_{a}=1-\frac{\dot{\varepsilon }}{{\rm{\Omega }}}{t}_{a}$$

In the experiment, the borosilicate specimen was compressed with a constant speed *V*, by a machine with a stiffness of *k*. In a typical speed controlled test the following condition applies

13 $$(\frac{\dot{\sigma }}{E}+\dot{\varepsilon })+\frac{\dot{\sigma }A}{kl}=\frac{V}{l}=R$$

where $\dot{\sigma }$ is stress rate, *E* is the modulus of the specimen, *A* and *l* are the cross-sectional area and length of the specimen. This equation can be further modified as

14 $$\dot{\sigma }=K(R-\dot{\varepsilon })$$

where

15 $$1/K=1/E+A/(k\cdot l)$$

By differentiating equation (11) and eliminating the stress rate using equation (14), one can get

16 $$\frac{\ddot{\varepsilon }}{\dot{\varepsilon }}=\frac{KR}{{\rm{\Phi }}}-\frac{K}{{\rm{\Phi }}}\dot{\varepsilon }-\frac{\psi \alpha }{{t}_{c}}\exp \{-{(\frac{{t}_{a}}{{t}_{c}})}^{\alpha }\}{(\frac{{t}_{a}}{{t}_{c}})}^{\alpha -1}{\dot{t}}_{a}$$

Together with equation (12), two first order differential equations in variables $\dot{\varepsilon }$ and *t*_a_ were thus obtained for the uniaxial compressive of borosilicate glass in supercooled liquid region.

It is noted that the characteristic equation of Eqs. 12 and 16 can be expressed as

17 $${\rm{\det }}[\begin{array}{cc}\lambda +\frac{KR}{S}-\frac{{\rm{\Gamma }}R}{{\rm{\Omega }}} & {\rm{\Gamma }}\frac{{R}^{3}}{{{\rm{\Omega }}}^{2}}\\ -\frac{1}{R} & \lambda +\frac{R}{\Omega }\end{array}]=0$$

where ${\rm{\Gamma }}=\psi \alpha {({t}_{a}/{t}_{c})}^{\alpha }\exp \{-{({t}_{a}/{t}_{c})}^{\alpha }\}$. By solving Eq. 17, one can obtain the corresponding characteristic eigenvalues

18 $$\lambda =\frac{R}{2S}[\mu \pm \sqrt{{\mu }^{2}-\omega }]$$

In which $\mu =-[K+{\rm{\Phi }}(1-{\rm{\Gamma }})/{\rm{\Omega }}]$, $\omega =4K{\rm{\Phi }}/{\rm{\Omega }}$. According to the linear stability theory, the instability could occur when the real part of the characteristic exponent becomes positive, i.e. when

19 $$K\le \frac{{\rm{\Phi }}}{{\rm{\Omega }}}({\rm{\Gamma }}-1)$$

This is a typical Hopf bifurcation^45^, and the emerging solution is a periodic oscillation.

Eqs 12 and 16 were further solved in Matlab with the parameters listed in Table 2. Here, the nominal Young’s modulus *E* is obtained from Fig. 1; the value of *k* used here is a typical stiffness of precision glass moulding machine^46^; *R* is obtained from equation (13) and ${\dot{\varepsilon }}_{0}$ is an initial reference strain rate which is used for calculating the initial value of *t*_a_ via Eq. (8); *t*_c_ is taken in the same order of bulk relaxation time ~ *η*/*G*, where *G* is the shear modulus. Stress sensitivity *Φ* is taken *via* ${\rm{\Phi }}=\partial \sigma /\partial \,\mathrm{ln}\,\dot{\varepsilon }=(\partial \sigma /\partial \dot{\varepsilon })(\partial \dot{\varepsilon }/\partial \,\mathrm{ln}\,\dot{\varepsilon })\approx 3\eta R$. Since it is hard to work out the exact values of *Ψ*, *t*_c_ and *Ω*, their values used here were obtained by fitting. It was noted that, increasing the values of *ψ*, α, *S*, *t*_c_, *Ω* can increase the amplitude of oscillation, and vice versa, but this does not affect the frequency; Increasing the value of *K* can decrease the amplitude and increase the frequency.

**Table 2:** Parameters used in the constitutive model for predicting the experimental result at 570 °C and loading speed 5 mm/min.

*E*(GPa)	*k* (N/m)	${{\bf{R}}}_{{\bf{0}}}$ (s^−1^)	*R* (s^−1^)	*Ψ*	*Φ* (MPa)	*α*	*t*_c_ (s)	Ω
0.5	10^8^	0.006	0.012	23.87	25.2	0.12	1	0.001

## Electronic supplementary material

supplementary information
