# Supplementary material for: Relaxation oscillation of borosilicate glasses in supercooled liquid region
Source: Sci Rep. 2017 Nov 20;7:15872. doi: 10.1038/s41598-017-16079-w (PMC5696471; doi:10.1038/s41598-017-16079-w)
Supplement: Supplementary file 1 — supplementary information [file 41598_2017_16079_MOESM1_ESM.doc]

**Relaxation oscillation of borosilicate glasses in supercooled liquid region**

W. D. Liu, L. C. Zhang^*^, K. Mylvaganam

*Laboratory for precision and nano processing technologies, School of Mechanical and Manufacturing Engineering, University of New South Wales, NSW 2052 Australia*

We have investigated the structural changes of borosilicate glass with the aid of ab initio simulations using the density functional theory (DFT). A sample having 160 atoms with composition 3Na_2_O-B_2_O_3_-6SiO_2_ (similar to L-BSL 7) and a mass density of 2.51 g/cm^3^ (same as experimental mass density) was simulated using DFT with the *ab initio* code SIESTA. The electronic structure was treated with the DFT using the Perdew-Burke-Ernzerhof (PBEsol)^1^ generalized gradient approximation (GGA) exchange correlation functional. A linear combination of atomic orbital basis sets with polarization functions were used for the description of valence electrons and norm-conserving nonlocal pseudo-potentials were used for atomic cores. The pseudopotential was constructed using the Troullier-Martins scheme^2^. The atoms were simulated with plane wave cut-off energy of 150.0 Ry. The Brillouin zone was sampled using a 3×3×3 Monkhorst-Pack k-grid^3^. The sample was heated to 4,500 K and equilibrated at 3,500, 2,500, 1,500, 825 and 625 K. The simulations were carried out until the temperature and the enthalpy were stabilized. As such the simulation time varies for different temperature simulations and for example at 825K the simulation length is ~8.5 ps. This is comparable to the time used in the work by Pedesseau et al.^4^

Figures 1a-1c shows the equilibrated structure of borosilicate glass at 3,500, 825 and 625 K. It is clear that when the sample was at 3,500 K (Fig. 1a), most of the atoms were either under coordinated or non-coordinated. On cooling the temperature gradually down to 825 K (Fig. 1b), the number of non-coordinated atoms decreased, and the structure of borosilicate glass is divided into borate-rich network (surrounded by dotted yellow line) and silicate-rich network. On further cooling down to 625 K (Fig. 1c), apart from the Na atoms (purple balls), most of the other atoms are either 3- or 4- coordinated. The partial pair distribution functions of B–O and Si–O pairs, calculated and compared at these temperatures (see Figures 1d-1e) clearly support this. For example, for the B–O pair (Figure 1d), the first peak locates at 1.34 Å at 3500 K, matching the distance of B-O bond in a trigonal borate. On reducing the temperature, at 825 K the first peak height decreased and a new large peak appeared at 1.49 Å, matching the distance of B-O bond in a tetrahedral borate. This indicates a change in the boron structure as observed by Pedesseau et al.^4^. At 625 K, both peaks become sharper because of glass transition. The small peak located at 1.6 Å should correspond to the distance between boron atoms and non-briging oxygens. For the Si - O pair, the location of the peak did not change significantly (Figure 1e). The trimodal distribution observed at 3,500 K should be due to thermal vibration.


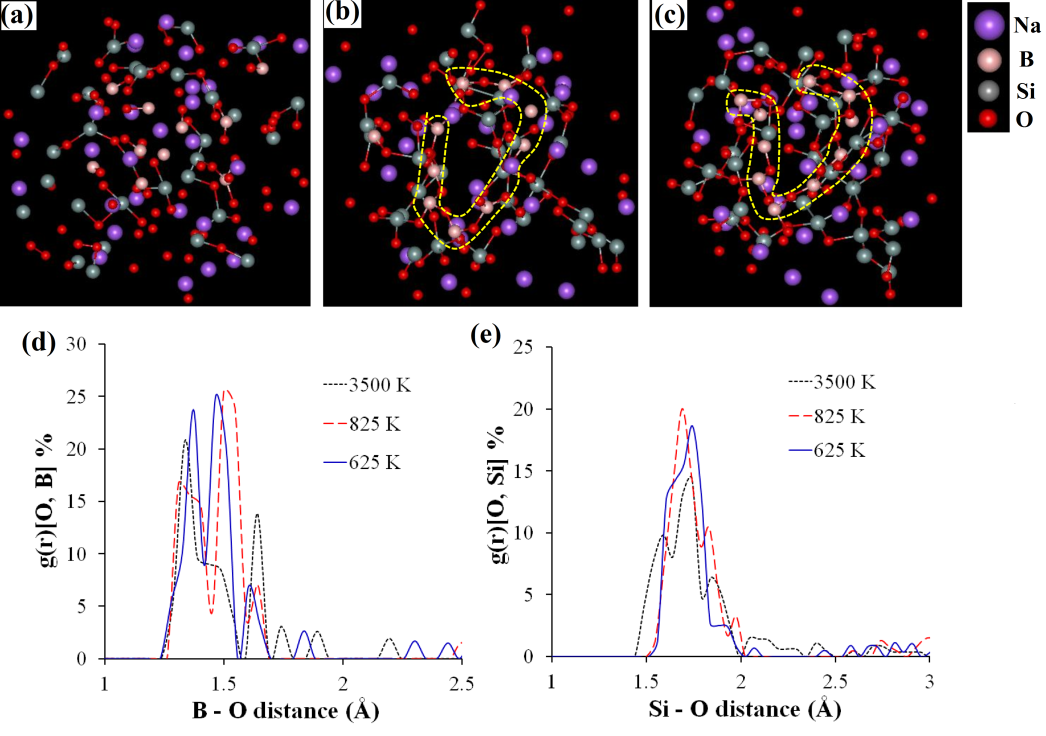


**Figure. 1.** Microstructure of borosilicate glass at (a) 3,500 K, (b) 825 K and (c) 625 K (H atoms are not shown for clarity), and the corresponding partial pair distribution functions of (d) B – O and (e) Si – O pairs.

**References**

1. Perdew, J. P., Burke, K. & Ernzerhof, M. Generalized gradient approximation made simple. *Phys Rev Lett* **77**, 3865-3868 (1996).
2. Troullier, N. & Martins, J. L. Efficient Pseudopotentials for Plane-Wave Calculations. *Phys Rev B* **43**, 1993-2006 (1991).
3. Monkhorst, H. J. & Pack, J. D. Special Points for Brillouin-Zone Integrations. *Phys Rev B* **13**, 5188-5192 (1976).
4. Pedesseau, L., Ispas, S. & Kob, W. First-principles study of a sodium borosilicate glass-former. II. The glass state. *Phys Rev B* **91**, 134202, (2015).
